# Association between Unmet Needs and Clinical Status in Patients with First Episode of Schizophrenia in Chile

**DOI:** 10.3389/fpsyt.2015.00057

**Published:** 2015-04-21

**Authors:** Natalia Jorquera, Rubén Alvarado, Nicolás Libuy, Valeria de Angel

**Affiliations:** ^1^Mental Health and Psychiatry Department North, Clinical Hospital of University of Chile, University of Chile, Santiago, Chile; ^2^Medicine Faculty, Public Health School, University of Chile, Santiago, Chile

**Keywords:** first episode of schizophrenia, needs assessment, mental health service, schizophrenia, unmet need

## Abstract

**Background:**

Schizophrenia is a severe mental disorder involving needs in several matters that are often not covered. A need is defined as a gap between the ideal state and the current state of a patient about a specific topic.

**Aim:**

To describe needs in patients with first episode of schizophrenia at the start of treatment and to describe associated clinical factors.

**Methods:**

Observational descriptive cross-sectional design. Patients were over 15 years old, with first episode schizophrenia, and admitted to treatment in the public health system from six districts in two cities of Chile, between 2005 and 2006. Sociodemographic data, clinical evaluations of current psychosis based on the Positive and Negative Syndrome Scale (PANSS), and the time of untreated psychosis were obtained. A clinical interview was carried out followed by the Camberwell Assessment of Need.

**Results:**

Twenty-nine patients were evaluated, 79.3% male, mean age 21.9 years old. The areas with more needs reported were; psychotic symptoms with 65.5% of sample, 21.1% of which reported it unmet; and daytime activities, where 44.8% of patients reported a need, 61.54% of them as unmet. The percentage of unmet needs correlated with PANSS score (*r* = 0.55; *p* = 0.003), and with time of positive symptoms prior to diagnosis (*r* = 0.416; *p* = 0.03).

**Discussion:**

Needs assessment in schizophrenia is necessary. It may affect its clinical course, be relevant in its management, and help monitor recovery. Defining the main needs in people with first episode schizophrenia and associated factors allows for a better design of treatment strategies in order to obtain better therapeutic results and recovery.

## Introduction

Schizophrenia is a severe mental disorder that begins in young people and creates great dysfunction in the social, occupational, and family environment. The lifetime prevalence rate worldwide of schizophrenia has been estimated at about 0.4%, while in Chile it has been estimated at 0.9% (1–3).

Schizophrenia carries great burden to those diagnosed and their families, as it is known to negatively affect key areas of functioning and lifestyle. People with schizophrenia are described as having less access to housing and employment; around two-thirds of patients never marry and maintain a reduced contact with family and friends. These social consequences of low productivity and having a reduced support group, coupled with the stigma attached to it, an increased risk of substance abuse and high medical comorbidity make this a very costly disease, both socially and economically (4–7).

To reduce the impact of schizophrenia, early diagnosis and treatment are paramount. When a first episode of psychosis appears, the earlier the treatment is received the more effective it may prove to be in the medium and long term (8–10). In light of this, and with the aim of shortening this critical period of untreated psychosis, the law in Chile guarantees the diagnosis and treatment of schizophrenia from the moment the disorder is suspected. This policy is known as GES (a Spanish acronym for Explicit Health Guarantees) (11).

In order to maximize the usefulness of interventions, they need to be not only timely but also effective. For this reason, interventions in first episode schizophrenia cannot be focused only on symptom control; it is also important to address the psychosocial dysfunction that occurs in the lives of each individual from the early stages of the disease (12, 13). For this, a proper evaluation of the needs of patients is required (14).

Needs are defined here as the difference between the optimal and the current state of the patient, in a specific field, and for which there is an effective intervention that could reduce this gap (15). The assessment of clinical and social needs has been proposed as an essential first step to the planning and implementation of patient-specific care plans (16). This approach makes for a more comprehensive intervention, tailored to people’s specific requirements, which ultimately helps achieve an acceptable level of independence and quality of life for these patients.

To help identify and specify needs, the Camberwell Assessment of Need (CAN) (14, 17) was developed. This instrument measures health needs of people with severe and enduring mental disorders such as schizophrenia on a 22-item scale. The CAN was designed to first enumerate the needs that a patient may have and, second, to ascertain whether these needs are met or unmet for this particular person. Unmet needs correspond to a problematic area where the necessary intervention that could improve the condition is not being received (15). A met need, on the other hand, is an area which a person identifies as problematic but is being addressed by their health or social services.

From a public health perspective, the issue of unmet needs can be defined at different levels (e.g., community and services), or from different perspectives (e.g., the patients, their families, or a clinicians) (9). Research has shown that professionals and service users have different perceptions of care needs. Points of view from health professionals and patients should be necessarily aligned to deliver rational interventions, and this should be taken into account in designing individualized care plans (18–20).

Studies show a strong inverse relationship between the number of unmet needs and quality of life in these patients, as well as its relation to symptom severity (21–23). This outlines the importance of interventions at the psychosocial level, in addition to the use of drugs.

Needs vary between different countries (23). In Chile, the needs of people in their first episode of schizophrenia have not been studied as has been for those at later stages of the disorder (24). In this context, the aim of this work is to measure needs in people with first episode schizophrenia and study the relationship between needs and clinical features of schizophrenia. As mentioned earlier, the time lag between first diagnosis and treatment initiation is a predictor of treatment outcome. For this reason, the time of untreated psychosis will also be examined to see if the number of needs varies according to this factor.

## Materials and Methods

### Participants

We included people who were 15 years old or more, and had a confirmed diagnosis of first episode schizophrenia. These people were being treated in the Psychiatric Services of the Public Health System in Chile.

People whose episode was secondary to another disease or due to another disorder (such as substance use, organic mental disorder, epilepsy, endocrine disorders, puerperal psychosis, etc.) were ruled out.

Recruitment took place in 2005 and 2006 from catchment areas of three hospitals, which comprised a total of six communes of the country. The Hospital Padre Hurtado encompasses the communes of La Granja and San Ramon, El Pino Hospital includes the communes of El Bosque and San Bernardo, and Iquique Hospital receives referrals from the communes of Alto Hospicio and Iquique.

### Assessment instruments

The CAN in its translated and validated version in Spanish was used to measure the number of present needs of subjects per area and the number of unmet needs (14, 17).

“Needs” are designated when a patient’s level of functioning in an area is below an established standard, and when there is also an effective intervention to correct the discrepancy. A need is considered met, or covered, when it has undergone the most effective intervention known to date; conversely, it is unmet when no intervention has been put in place, or when there was an intervention but other more effective interventions exist, and so the need was not satisfied (18).

Camberwell Assessment of Need is an interview comprising 22 items grouped into five conceptual domains; health, consisting of physical health, psychotic symptoms, drugs, alcohol, safety to self, safety to others, and psychological distress; basic needs, consisting of food, daytime activities, and accommodation; social needs, consisting of sexual expression, intimate relationships, and company; service needs, consisting of information on condition and treatment, telephone (having access to a telephone), transport, and benefits; and functioning, consisting of basic education, money, child care, self-care, and looking after the home. The instrument maintains an identical structure for all areas, and each area of need includes four sections. The first section establishes whether there is a current problem in the area, constituting a filter before further exploring this area. If the patient does not consider that area to be problematic – so does not identify that item as a need – no ratings are required and the interviewer continues to the following item. If the patient does identify that item as a need, they are required to rate it. Each item is rated on a scale of three conditions: 0 = no problem, 1 = absent or moderate problem due to the person receiving ongoing interventions (met need), 2 = actual serious problem and no interventions received (unmet need) (17, 25).

Symptoms of schizophrenia were assessed with the Positive and Negative Syndrome Scale (PANSS). The PANSS is a widely used medical scale used for measuring symptom severity of patients with schizophrenia. It comprises three subscales that cover positive symptoms, negative symptoms, and general psychopathology (26, 27).

Sociodemographic variables, such as sex, age, marital status, who they live with, educational level, employment status, support network (sporting, religious, and social participation), were obtained by means of a questionnaire.

### Design and procedure

A cross-sectional study was carried out based on an assessment of people with a diagnosis of first episode of schizophrenia with the CAN, PANSS instrument, and a sociodemographic survey.

The cases were detected through GES admission records. Case confirmation was achieved through information from the treating team (clinical records or an interview with the physician).

Thirty-three cases were identified, two of them could not be located (they had abandoned treatment), one refused to be surveyed, and one moved to another region of the country and failed to be surveyed. The interview took place at the outpatient psychiatric unit where they were being treated – mobilization costs were paid for – and the interviewer carried out the survey. The interviewers were professionals (two occupational therapists and one psychologist) specially trained for this activity.

The time lag between suspicion of the first episode of schizophrenia in primary care by a general practitioner and confirmation of the diagnosis and initiation of treatment by a psychiatrist was also recorded.

The period in months of depressive, positive, and negative symptoms before diagnosis was recorded.

### Statistical analysis

A description of the sample was performed according to sociodemographic variables, symptoms of schizophrenia, number of needs of individuals, and number and percentage of unmet needs. The most frequent areas of needs were also described.

We analyzed whether there was a correlation between the percentage of unmet needs for patients and their clinical state according to the PANSS as well as between the percentage of unmet needs and time of untreated psychosis. Untreated psychosis was measured as time between start of the first depressive, positive, and/or negative symptoms and diagnosis of episode. A Pearson’s correlation analysis between unmet needs as a continuous percentage variable with values between 0 and 1, and the PANSS score for each of the subscales and total score as continuous variables was performed. Likewise, a Pearson correlation between unmet needs and untreated psychosis time (as a continuous variable measured in months) was performed. Bilateral confidence levels of 95% were considered. Correlations between these needs and the PANSS score were compared before and after excluding the need “psychotic symptoms” as there is a possibility of an overlap between this need and the PANSS. This was done to avoid a possible bias.

Database editing, description, and statistical analysis were performed using R 3.0.1 software.

### Ethical considerations

Before interviewing the patients, this project was reviewed and approved by the Ethics Committee of the Faculty of Medicine, University of Chile. Patients were informed about the study and informed consent was obtained.

## Results

### Demographics

The sample consisted of 29 patients, of whom 23 were men (79.3%). The mean age was 21.9 years with a standard deviation of 6.1 years, and the median was 20 years. The patients’ marital status indicated that 24 patients were single (82.7%), 2 were married (6.8%), 2 cohabited (6.8%), and 1 was separated (3.4%). There was a median of five people living under the same roof as the patient.

Regarding employment, nine patients were students at the time of the interview (31.0%), four had occasional work (13.7%), six had stable jobs (20.6%), seven patients were unemployed (24.1%), and three patients were classified as “other” (10.3%).

In respect to the level of studies prior to the diagnosis, the mean number of years of education completed was 10.2 years with an SD of 2.2, and a median of 10 years.

### Areas of need

In the sample studied, we found that the five most frequent areas of present needs in patients with first episode psychosis were psychotic symptoms, daytime activities, information on condition and treatment, company, and self-care. Self-care and psychotic symptoms were the two areas with the highest percentage of met needs, while daytime activities was the area with the highest percentage of unmet needs. Areas that did not report needs were: physical health, basic education, telephone, and benefits. An overview of the needs is presented in Table 1.

**Table 1:** **Identified and unmet needs in people with first episode of schizophrenia in three centers in Chile (*n* = 29)**.

Needs	With need		Unmet need	
	*n*	%	*n*	%
Accommodation	1	3.5	1	100
Food	3	10.3	1	33.3
Looking after the home	1	3.5	0	0
Self-care	6	20.7	0	0
Daytime activities	13	44.8	8	61.5
Physical health	0	0	–	–
Psychotic symptoms	19	65.5	4	21.1
Info on condition and treatment	11	37.9	4	36.3
Psychological distress	4	13.8	0	0
Safety to self	5	17.2	3	60
Safety to others	1	3.5	1	100
Alcohol	4	13.8	1	25
Drugs	1	3.5	1	100
Company	11	37.9	4	36.4
Intimate relationships	3	10.3	0	0
Sexual expression	4	13.8	1	25
Child care	2	6.9	0	0
Basic education	0	0	–	–
Telephone	0	0	–	–
Transport	3	10.3	0	0
Money	3	10.3	1	33.3
Benefits	0	0	–	–

With respect to patient needs, there is a mean of 3.3 areas of need per patient, with an SD of 2.1 and a median of 3 areas of needs.

As for the unmet needs, the mean was 1.0 unmet need per patient, with an SD of 1.2, and a median of 1. Table 2 shows how many patients expressed a certain number of needs.

**Table 2:** **The amount of patients per quantity of needs (*n* = 29)**.

(A) No. of needs	No. of subjects (%)	(B) No. of unmet needs	No. of subjects (%)
0	2 (6.8)	0	12 (41.3)
1	3 (10.3)	1	9 (31)
2	9 (31)	2	5 (17.3)
3	3 (10.3)	3	2 (6.8)
4	4 (13.7)	4	0 (0)
5	3 (10.3)	5	1 (3.4)
6	2 (6.8)	6 or more	0 (0)
7	2 (6.8)		
8	1 (3.4)		
9 or more	0 (0)		

### Clinical status

Information on the clinical status of patients at the time of the interview was obtained by applying the PANSS. Table 3 shows the mean total PANSS score for this sample, as well as the mean scores in each subscale.

**Table 3:** **Mean and median PANSS scores in people with first episode of schizophrenia in three centers in Chile (*n* = 29)**.

Results in PANSS	Mean ± SD	Median
Positive symptoms	16.31 ± 14.17	12
Negative symptoms	25 ± 13.39	22
General psychopathology	44.66 ± 25.59	34
Total PANSS	85.97 ± 51.1	67

### Time of untreated psychosis

There was a mean of 4.7 months of depressive symptoms before diagnosis, with an SD of 3.4 and a median of 5 months. Negative symptoms appeared a mean of 5.8 months prior to formal diagnosis, with an SD of 3.2, and a median of 7 months. Positive symptoms, on the other hand, appeared a mean of 4.4 months before a diagnosis, with an SD of 2.7, and a median of 6 months.

### Correlation between needs and clinical state

We explored whether there was correlation between unmet needs and clinical state as measured by the PANSS. The time of untreated psychosis was also explored.

We found a significant positive correlation between the percentage of unmet needs and total PANSS scores with a Pearson correlation coefficient of *r* = 0.55 (CI: 0.22–0.77, *p* < 0.01), such that, the higher the PANSS score, the higher the number of unmet needs reported by the patient. This correlation is maintained when analyzing the three PANSS subscales individually; the negative symptom subscale, *r* = 0.52 (CI 0.18–0.75, *p* < 0.01), the positive symptom subscale *r* = 0.52 (CI: 0.17–0.75, *p* < 0.01), and general psychopathology *r* = 0.54 (CI 0.21–0.77, *p* < 0.01).

When the area of need of psychotic symptoms is excluded and the percentage of unmet needs is correlated with PANSS scores, there is a trend to keep the positive correlation; however, the correlation is non-significant for the total scores (*r* = 0.35; *p* = 0.08) as well as any of the subscales of positive symptoms (*r* = 0.37; *p* = 0.06), negative symptoms (*r* = 0.33; *p* = 0.09), and general psychopathology (*r* = 0.31; *p* = 0.12).

Moreover, we found a significant correlation between the percentage of unmet needs and time of positive symptoms prior to diagnosis, *r* = 0.42 (CI: 0.04–0.69, *p* = 0.03). Therefore, the longer the time elapsed between the first appearance of positive symptoms and a diagnosis (and therefore treatment), the higher the reported number of unmet needs.

In this case, when the area of need of psychotic symptoms is excluded and the correlation of percentage of unmet needs is evaluated against time of positive symptoms prior to diagnosis, the significant correlation remains; *r* = 0.49 (CI 0.12–0.74, *p* = 0.012).

When exploring other correlations between percentage of unmet needs and time of depressive symptoms (*r* = 0.12; *p* = 0.57), and negative symptoms before diagnosis (*r* = 0.07; *p* = 0.71), no significant correlation were found.

## Discussion

This study found that the main needs identified by patients with first episode of psychosis were in the areas of psychotic symptoms, daytime activities, information on condition and treatment, company, and self-care. The fact that most patients had the area of psychotic symptoms as a met need must be emphasized. Other areas in which the majority of patients perceived their needs to be met were in: information on condition and treatment, company, and self-care. The area of daytime activities, on the other hand, was found by most to be unmet.

It should be noted that in these patients, one of the most important met needs was psychotic symptoms, while daytime activities were a major need that was largely reported as unmet. This can be easily understood if we consider that treatment is usually aimed at symptomatic control with psychotropic drugs even when the condition is not confirmed but suspected. This clinical element receives more attention and care in these patients, at the cost of neglecting an equally important aspect for functionality such as activities of daily living. It is this aspect that has a major role in psychosocial interventions. There is strong evidence that psychoeducational family interventions, social skills training, and employment support combined with medication are associated with a significantly lower risk of relapse compared to drug treatment alone (9, 13).

Another important result is the correlation between unmet needs and symptoms measured by PANSS. This result supports the hypothesis that a larger number of unmet needs are associated with a decline in the subject’s clinical condition as measured by the PANSS. This correlation, however, is lost when excluding psychotic symptoms as a need. A possible interpretation following from this result is that by evaluating this particular need, one is essentially measuring the same phenomenon as the clinical evaluation. An alternative explanation could be that there was insufficient power to detect a result due to the small sample size of this study.

When the association between time of untreated psychosis and needs was analyzed, a significant positive correlation between time of positive symptoms and unmet needs was found. That this association remained after excluding the need psychotic symptoms confirms the importance of diagnosis and early intervention.

The current results support previous literature in that very similar areas of need were identified. Studies from other countries also state that psychotic symptoms are a frequently reported need (22, 23, 28).

Ochoa et al. reported needs in patients with schizophrenia in Spain. They detected the most frequent needs for patients as being: psychotic symptoms, looking after the home, food, and information on condition and treatment. They found the results of the PANSS to show a positive correlation with unmet needs, such that patients with more severe clinical symptoms and high disability had a larger number of unmet needs (22).

Rosales Varo and colleagues also studied needs in patients diagnosed with schizophrenia in Spain. After asking patients and therapists, they concluded that the needs related to clinical symptoms such as psychotic symptoms and anxiety appeared most frequently. The average number of needs identified was 6.5, the most frequent of which were daytime activities, company, psychotic symptoms, anxiety, basic education, and money management. The sociodemographic variables most associated with these needs were not having a partner, low educational level, and receiving a disability pension. Patients with lower educational levels and those with a disability pension showed a greater number of needs (29).

In Chile, Alvarado and colleagues measured needs in 141 patients with schizophrenia or related psychotic disorders who had been undergoing treatment for 2 to 4 years. They identified socioeconomic variables, time since diagnosis, course of the disease, and clinical state at the time of the interview, and found them to be associated with the number of unmet needs. The number of unmet needs was significantly associated with functionality and PANSS score. Additionally, they found an indirect association with patients who identified with an ethnic minority, number of people living with the patient, number of years of schooling, patient age at diagnosis, and an annual relapse rate (24).

Unlike these studies (22, 24, 29), which used samples of patients who had had a diagnosis of schizophrenia or a related psychotic disorder for some time, the current investigation looks at first episode schizophrenia.

In the study of Alvarado et al. (24), we can see that the number of unmet needs after 2–4 years of treatment of the first episode of schizophrenia is similar to our research; the mean number of needs reported per person is 1 and 1.2, respectively. Furthermore, the percentage of patients with unmet needs is also similar; with 58.7 and 54.6% having at least one area of unmet needs, respectively. From these data, one could think that if these unmet needs remain after 2–4 years of treatment, care services are not being able to provide effective cover for them. This hypothesis should be tested in future studies to propose improvements in addressing unmet needs from the first episode of schizophrenia.

It seems important to highlight that considering the patient’s perspective when assessing needs is crucial to designing appropriate treatment approaches in patients with schizophrenia. Meeting the expressed demands of people with schizophrenia may offer better clinical outcomes, quality of life, functionality, and reduce the burden of disease. Individualized clinical and psychosocial exploration of the problematic areas as well as the concordance between patients and their significant others with the therapeutic team is crucial in their recovery (19, 20). Thus, research on the needs of patients from their own perspective is very important for their recovery, designing individual treatment plans, and the planning of mental health services at the population level.

Among the strengths of this study, we highlight the fact that it is the first experience in Chile on measuring needs after a first psychotic episode. Further strengths include the measurement of clinical condition using the PANSS, and recording time of untreated psychosis in different treatment referral centers in the country. An important limitation was the small number of cases. Future work should focus on expanding the number of cases and improve the statistical power to allow for further variables being added to the analysis. Another important limitation is that the association between the clinical status and needs can be measures of the same clinical phenomenon, as had discussed above. This point, however, may be better understood only with further research development in this area.

In conclusion, this paper has presented the main needs in patients with first episode schizophrenia and considered its relationship to symptoms and the time before diagnosis. An association between unmet needs and PANSS scores was found as well as an association between unmet needs and time of untreated positive symptoms. Studying needs in subjects with first episode of schizophrenia and associated factors may help to design treatment strategies in order to obtain better therapeutic results and recovery.

## Conflict of Interest Statement

The authors declare that the research was conducted in the absence of any commercial or financial relationships that could be construed as a potential conflict of interest.
